# LncRNA FOXD3-AS1 promoted chemo-resistance of NSCLC cells via directly acting on miR-127-3p/MDM2 axis

**DOI:** 10.1186/s12935-020-01402-9

**Published:** 2020-07-29

**Authors:** Zhaolong Zeng, Guofang Zhao, Huangkai Zhu, Liangqin Nie, Lifeng He, Jiangtao Liu, Rui Li, Shuai Xiao, Gang Hua

**Affiliations:** 1 Department of Thoracic Surgery, Hwamei Hospital, University of Chinese Academy of Sciences, No. 41 Northwest Street, Haishu District, Ningbo City, 315000 Zhejiang Province China; 2 Department of Thoracic Surgery, Ningbo Institute of Life and Health Industry, University of Chinese Academy of Sciences, Ningbo, Zhejiang Province, China; 3 School of Medicine, Ningbo University, Ningbo, Zhejiang Province, China; 4 Department of Radiology, Hwamei Hospital, University of Chinese Academy of Sciences, Ningbo, Zhejiang Province, China; 5 Department of Spinal Surgery, Hwamei Hospital, University of Chinese Academy of Sciences, Ningbo, Zhejiang Province, China

**Keywords:** Chemo-resistance, NSCLC, FOXD3-AS1, miR-127-3p, MDM2, Cisplatin

## Abstract

**Background:**

This study aims to investigate the mechanism underlying the high level of long non-coding RNA FOXD3-AS1 in cisplatin-resistant NSCLC cells.

**Methods:**

Cisplatin-resistant cells were generated from A549 cells. CCK-8 were used to evaluate cell proliferation. The FOXD3-AS1, miR-127-3p, MDM2 and MRP1 mRNA expression levels were confirmed by qRT-PCR. Protein levels of MDM2 and MRP1 were determined by western blot assay. Luciferase reporter and RNA pull-down assays were evaluated the relationship between miR-127-3p and FOXD3-AS1/MDM2. In vivo tumor growth was evaluated in a xenograft nude mice model.

**Results:**

FOXD3-AS1 was up-regulated in cisplatin-resistant NSCLC cells (A549/DDP and H1299/DDP cells) in comparison with their parental cell lines. Overexpression of FOXD3-AS1 promoted cisplatin-resistance in A549 and H1299 cells; while FOXD3-AS1 knockdown sensitized A549/DDP and H1299/DDP cells to cisplatin treatment. FOXD3-AS1 regulated miR-127-3p expression by acting as a competing endogenous RNA, and miR-127-3p repressed MDM2 expression via targeting the 3′UTR. MiR-127-3p overexpression and MDM2 knockdown both increased the chemo-sensitivity in A549/DDP cells; while miR-127-3p knockdown and MDM2 overexpression both promoted chemoresistance in A549 cells. Further rescue experiments revealed that miR-127-3p knockdown or MDM2 overexpression counteracted the suppressive effects of FOXD3-AS1 knockdown on chemo-resistance and MRP1 expression in A549/DDP cells. In vivo studies showed that FOXD3-AS1 knockdown potentiated the antitumor effects of cisplatin treatment. Inspection of clinical samples showed the upregulation of FOXD3-AS1 and MDM2, and down-regulation of miR-127-3p in NSCLC tissues compared to normal adjacent tissues.

**Conclusion:**

In conclusion, our results suggest that LncRNA FOXD3-AS1 promotes chemo-resistance of NSCLC cells via directly acting on miR-127-3p/MDM2 axis. Our findings may provide novel perspectives for the treatment of NSCLC in patients resistant to chemotherapy.

## Background

Lung cancer represents one of the most deadly tumor malignancies with patients having very low overall survival rate, due to the high metastasis of this disease [1, 2]. Non-small cell lung cancer (NSCLC) is the main type of lung and accounts for more than 80% of all the lung cancer cases [3, 4]. Chemotherapy and surgical resection are the main strategies for treating NSCLC, and cisplatin-based chemotherapy has been widely used in treating NSCLC [3]. However, the development of cisplatin resistance has been a major obstacle in treating NSCLC [5, 6]. Unfortunately, the factors that contribute to cisplatin resistance in NSCLC have not been fully understood yet. Hence, exploration of novel strategies to promote cisplatin sensitivity is urgent for us to have a better control of this malignancy.

Long non-coding RNAs (lncRNAs) belong to a class of non-coding RNAs with > 200 nucleotides in length, and various biological functions of lncRNAs such as cell proliferation, cell apoptosis, cell differentiation have been identified [7, 8]. Emerging evidence has emphasized the vital roles of lncRNAs in the regulation of cisplatin resistance in NSCLC. For examples, lncRNA colon cancer associated transcript 1 (CCAT1) was up-regulated in cisplatin-resistant NSCLC cells, and CCAT1 regulated SRY-Box 4 expression to promote cisplatin resistance in NSCLC cells [9]. LncRNA bladder cancer associated transcript 1 (BLCAT1) was up-regulated in NSCLC cells and BLACAT1 overexpression contributed to NSCLC cisplatin resistance via modulating autophagy [10]. LncRNA EGFR antisense RNA 1 (EGFR-AS1) was found to be elevated in plasma of NSCLC patients and EGFR-AS1 overexpression promoted cisplatin resistance in NSCLC [11]. Recent evidence implied that lncRNA FOXD3 antisense RNA1 (FOXD3-AS1) was involved in the progression of different malignancies [12–15]. Unfortunately, little is known about FOXD3-AS1 regarding chemo-resistance in NSCLC.

One of the main functions of lncRNA is known to act as a competing endogenous RNA (ceRNA) to influence microRNAs (miRNAs) expression [16]. MiRNAs belong to a class of small non-coding RNAs with 21–23 nucleotides in length and play pivotal roles in regulating cellular functions [16–18]. There is existing evidence showing that miR-127-3p acts as a tumor suppressor in various types of cancers including ovarian cancer [19], osteosarcoma [20], glioblastoma [21] and oral squamous cell carcinoma [22]. Up to date, the role of miR-127-3p in NSCLC progression and chemo-resistance has not been explored yet.

In this study, we analyzed the FOXD3-AS1 in cisplatin-resistant NSCLC cell lines (A549/DDP and H1229/DDP cells) and explored the underlying mechanisms of FOXD3-AS1 in regulating cisplatin resistance in A549/DDP and H1229/DDP cells. This study may provide novel perspectives for the treatment of NSCLC in patients resistant to chemotherapy.

## Materials and methods

### Collection of clinical samples

The procedures for collecting patients’ lung tissue samples were approved by the Ethics Committee of Hwamei Hospital, and the written informed consent was signed by each patient. The lung cancer tissues and normal adjacent lung tissues were collected from 40 NSCLC patients who received surgical resection at Hwamei Hospital between March 2015 and November 2017. All the patients had no chemo-/radio-therapy before the surgery. The collected tissue samples were immediately frozen in liquid nitrogen and stored at − 80 °C for future use.

### Cell lines and culture

Normal human lung bronchial epithelial cells (NHBE) and NSCLC cell lines (A549 and H1299) were purchased from the ATCC (Manassas, USA). The NHBE, A549 and H1299 cells were cultured in DMEM (Gibco, Grand Island, USA) supplemented with 10% fetal bovine serum (FBS; Invitrogen, Carlsbad, USA), 100 mg/ml streptomycin and 100 U/ml penicillin in a humidified air at 37 °C with 5% CO_2_. To establish cisplatin-resistant A549 (A549/DDP) and H1299 cells (H1299/DDP), A549 and H1299 cells were first treated with 0.1 mg/ml cisplatin (Tocris Bioscience, Minneapolis, USA) and then gradually exposed to cisplatin with step-wise increased concentrations till 1 mg/ml. The established A549/DDP and H1299/DDP cells were cultured in full medium containing 1 mg/ml until further use.

### Cell transfections with plasmids, miRNAs and small interfering (siRNAs)

The control vector (pcDNA3.1) and the vector for overexpressing FOXD3-AS1 (pcDNA3.1-FOXD3-AS1) or murine double minute 2 (MDM2; pcDNA3.1-MDM2) were purchased from GenePharma (Shanghai, China). The mimics and inhibitors for miR-127-3p and the negative controls were purchased from Ribobio (Guangzhou, China). The siRNAs for targeting FOXD3-AS1 (si-FOXD3-AS1) or MDM2 (si-MDM2), and the respective scrambled negative controls were designed and synthesized by Ribobio. All the relevant oligonucleotides sequences were shown in Additional file 1: Table S1. All the plasmids, miRNA and siRNA oligonucleotides were transfected into NSCLC cells using Lipofectamine 2000 reagent (Invitrogen). The time point for harvesting the transfected cells was at 48 h post-transfection.

### Cisplatin treatment and cell counting kit-8 (CCK-8) assay

The transfected NSCLC cells were seeded onto the 96-well plates. Following an incubation of 24 h, cells were incubated with different concentrations (0, 3, 10, 30, 100 and 300 μM) of cisplatin for 48 h. After cisplatin treatment, the NSCLC cells were further incubated with CCK-8 solution as per manufacturer’s protocol for 1 h at 37 °C. Cell viability of the transfected cells were determined by measuring the absorbance at 450 nm. IC50 were determined as the concentration of cisplatin that produced 50% inhibition on cell viability. Higher IC50 values indicate increased chemo-resistance.

### Quantitative real-time PCR

Total RNA was extracted from transfected cells or tissues by using TRIzol reagent (Takara) and was quantified with the Nanodrop 2000 machine (Wuhan Bonnin Technology Ltd., Wuhan, China). The cDNA was synthesized from 1 µg purified RNA using the PrimeScript RT-PCR kit (Takara). The quantification of the FOXD3-AS1, miR-127-3p, MDM2 and multidrug resistance protein 1 (MRP1) was performed on the ABI7900 real-time PCR detection system (Applied Biosystems) using SYBR Premix Ex Taq II kit (Takara) or TaqMan assay kit (Takara) by following the detailed instructions from the supplier. Comparative Ct method was utilized to detect the expressions of FOXD3-AS1, miR-127-3p, MDM2 and MRP1. U6 was used as internal control for miR-127-3p expression, and GAPDH was used as internal control for FOXD3-AS1, MDM2 and MRP1 expressions. The primers for respective genes were as follows: FOXD3-AS1, F:5′-GGTGGAGGAGGCGAGGATG-3′ and R: 5′-AGCGGACAGACAGGGATTGG-3′; miR-127-3p, F:5′-GGAAGATCTGTAGTCCTGTCTGTTGGTCAG-3′ and R: 5′-CCCAAGCTTCCTGAAGAACTGCTTCCGCC-3′; MDM2, F:5′- GGCTCTGTGTGTAATAAGGGAGA-3′ and R:5′- GGACTGCCAGGACTAGACTTTG-3′; MRP1, F:5′-ACCCTAATCCCTGCCCAGAG-3′ and R: 5′-CGCATTCCTTCTTCCAGTTC-3′. U6, F:5′-GCCATACCACCCTGAACG-3′ and R:5′-TGCAGGGTCCGAGGTATTCG-3′; GAPDH, F:5′-AAGGTGAAGGTCGGAGTCAAC-3′ and R:5′-GGGGTCATTGATGGCAACAATA-3′.

### Western blot analysis

Total proteins from cells were extracted using RIPA lysis buffer (GBCBIO Technologies, Lnc., Guangzhou, China) supplied with protease inhibitors (Roche, Basel, Switzerland). Equal amounts of extracted proteins were separated on a 10% SDS-PAGE and were then transferred to the PVDF membranes (Sigma, St. Louis, USA). The PVDF membranes were then incubated with 5% non-fat milk in tris-buffered saline with Tween 20, and the blocked membranes were subjected to primary antibodies incubation for overnight at 4 °C. These primary antibodies include MDM2 (1:2000; Cell Signaling Technology, Danvers, USA), MRP1 (1:1500; Cell Signaling Technology) and β-actin (1:2000; Cell Signaling Technology). After a further washing with TBST, membranes were again incubated with horseradish peroxidase-conjugated secondary antibodies (1:2500; Cell Signaling Technology). The western blot bands were visualized by ECL Detection kit (HANNOTECH Biosciences, Dongguan, China) by following the supplier’s instruction.

### Luciferase reporter assay

Online database StarBaseV3.0 was performed to predict putative binding sites for miR-127-3p. The wild type or mutant (MUT) fragments for FOXD3-AS1 and MDM2 3′ untranslated region (3′UTR) were amplified with PCR and were inserted into the pGL3 luciferase reporter vector (Promega, Madison, USA) to produce different reporter vectors including FOXD3-AS1 (WT), MDM2 3′UTR (WT), FOXD3-AS1 (MUT) and MDM2 3′UTR (MUT). The A549/DDP cells were co-transfected with reporter vectors (FOXD3-AS1 (WT), MDM2 3′UTR (WT), FOXD3-AS1 (MUT) and MDM2 3′UTR (MUT)) and miRNA oligonucleotides using Lipofectamine 2000 reagent. At 48 h after transfection, A549/DDP cells were harvested and the relative luciferase activity was determined using Dual-Luciferase Reporter Assay System (Promega).

### RNA pull-down assay

The RNA pull-down assay was carried out to examine the interaction between FOXD3-AS1 and miR-127-3p. Briefly, the biotinylated FOXD3-AS1 (WT), FOXD3-AS1 (MUT) and negative control probes were synthesized by RiboBio. The RNA pull-down assay was performed using the Magnetic RNA Pull-Down Kit (Thermo Fisher Scientific) according to the supplier’s instruction. Cells were transfected with biotinylated probes and the cell lysates were incubated with the M-280 streptavidin magnetic beads (Invitrogen). The miR-127-3p expression level was measured by qRT-PCR.

### Animal in vivo experimental assays

The male BALB/c nude mice with specific-pathogen-free grade were purchased from Zhejiang Laboratory Animal Center (Hangzhou, China). All the animals had free access to food and water. The experimental procedures for the animal studies were approved by the Animal Ethics Committee of Hwamei Hospital. For the in vivo tumor growth assay, the acclimatized animals were injected subcutaneously with A549/DDP cells stabled transfected with FOXD3-AS1 knockdown lentiviral vector (sh_FOXD3-AS1) or control vector (sh_NC). When the tumor size reached ~ 60 mm^3^, mice were treated with cisplatin (5 mg/kg, intraperitoneal) or normal saline (2 ml/kg, intraperitoneal) twice per week for 3 weeks. The growth of the tumors was determined for 5 weeks on a weekly basis after initial injection of NSCLC cells. At the end of the experiment, mice were killed and tumor tissues were harvested for weighting and gene expression detection.

### Statistical analysis

The statistical analysis was carried out using GraphPad Prims 6.0 Software (GraphPad Prism, La Jolla, USA). The data collected from the experimental assays were presented as mean ± standard deviation. The statistical significance was evaluated with unpaired Student’s *t* test or one-way ANOVA followed with Turkey’s post hoc test. P < 0.05 was considered to be statistically significant.

## Results

### FOXD3-AS1 promoted chemo-resistance in NSCLC cells

**Fig. 1:**
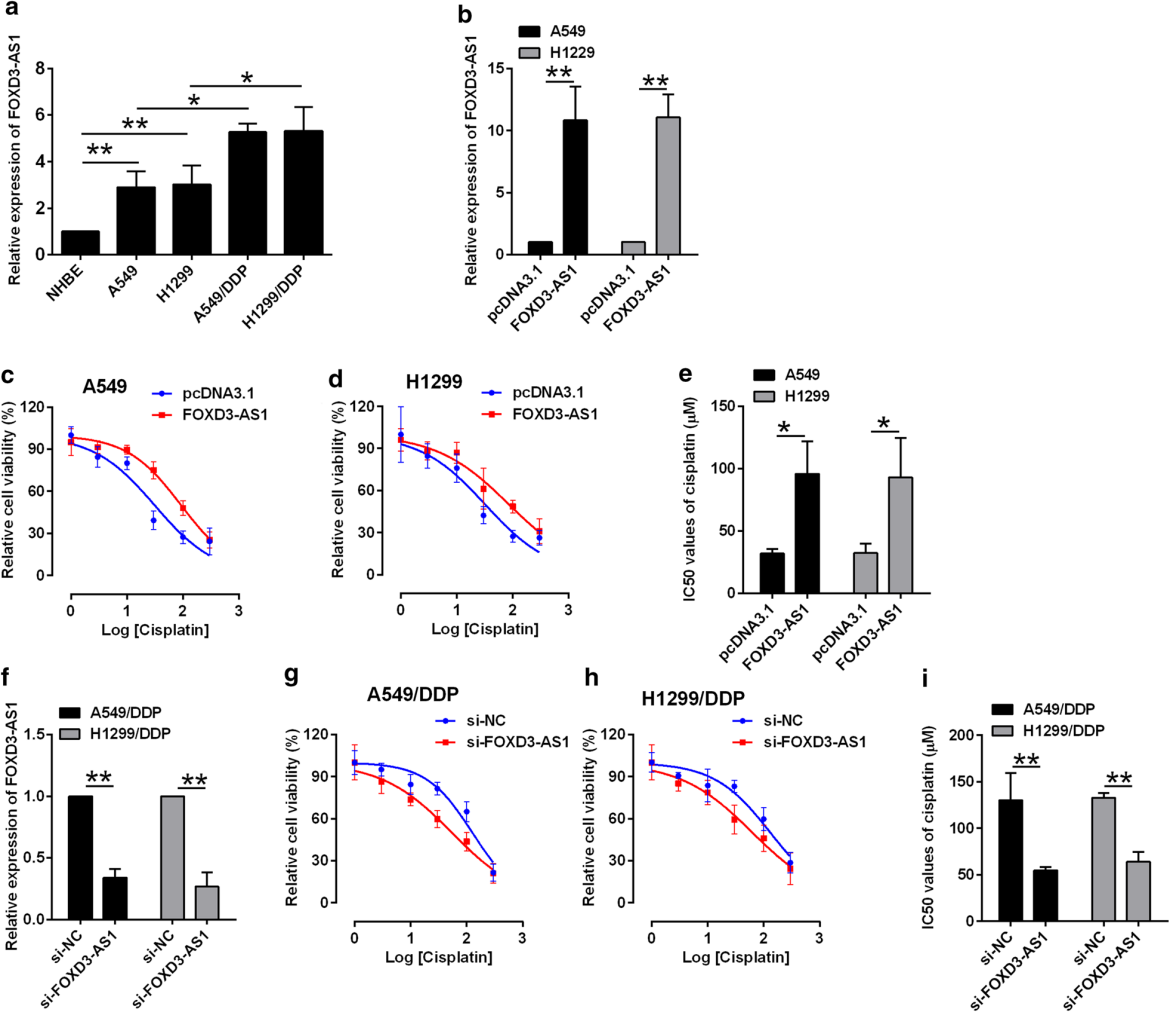
FOXD3-AS1 promoted chemo-resistance in NSCLC cells. **a** qRT-PCR determination of FOXD3-AS1 in cell lines including NHBE, A549, H1299, A549/DDP and H1299/DDP. **b** qRT-PCR determination of FOXD3-AS1 in A549 and H1299 cells with pcDNA3.1 or pcDNA3.1-FOXD3-AS1 transfection. **c**–**e** CCK-8 assay determination of IC50 values (cisplatin) in A549 and H1299 cells with pcDNA3.1 or pcDNA3.1-FOXD3-AS1 transfection. **f** qRT-PCR determination of FOXD3-AS1 in A549 and H1299 cells with si-NC or si-FOXD3-AS1 transfection. **g**–**i** CCK-8 assay determination of IC50 values (cisplatin) in A549 and H1299 cells with si-NC or si-FOXD3-AS1 transfection. N = 3 biological samples, and each sample was assayed in triplicates. Significant different between different treatment groups were shown as *P < 0.05 and **P < 0.01

The expression of FOXD3-AS1 was compared in both NSCLC cells and DDP-resistant NSCLC cells. The FOXD3-AS1 expression was up-regulated in NSCLC cell lines (A549 and H1299) in comparison with NHBE cells (Fig. 1a), and further comparison showed that FOXD3-AS1 expression was up-regulated in DDP-resistant cell lines (A549/DDP and H1299/DDP) in comparison with their parental cells lines, respectively (Fig. 1a). The cell viability of the A549 and H1299 by pcDNA3.1 or pcDNA3.1-FOXD3-AS1 was determined by CCK-8 assay. The transfection with pcDNA3.1-FOXD3-AS1 in A549 and H1229 cells drastically increased FOXD3-AS1 expression in comparison with pcDNA3.1 transfection (Fig. 1b), and FOXD3-AS1 overexpression attenuated cisplatin-induced cell inhibition and significantly increased IC50 values of cisplatin in A549 and H1299 cells (Fig. 1c–e), suggesting that FOXD3-AS1 overexpression promotes cisplatin-resistance in A549 and H1299 cells. In contrast, FOXD3-AS1 siRNA transfection (si-FOXD3-AS1) caused a significant decrease in the FOXD3-AS1 expression of A549/DDP and H1299/DDP cells (Fig. 1f), and by determining the cell viability, the results revealed that FOXD3-AS1 knockdown decreased the IC50 for cisplatin (Fig. 1g–i), suggesting that FOXD3-AS1 inhibition sensitizes A549/DPP and H1299/DPP cells to cisplatin treatment.

### FOXD3-AS1 regulates DDP-resistance in NSCLC cells via repressing miR-127-3p

**Fig. 2:**
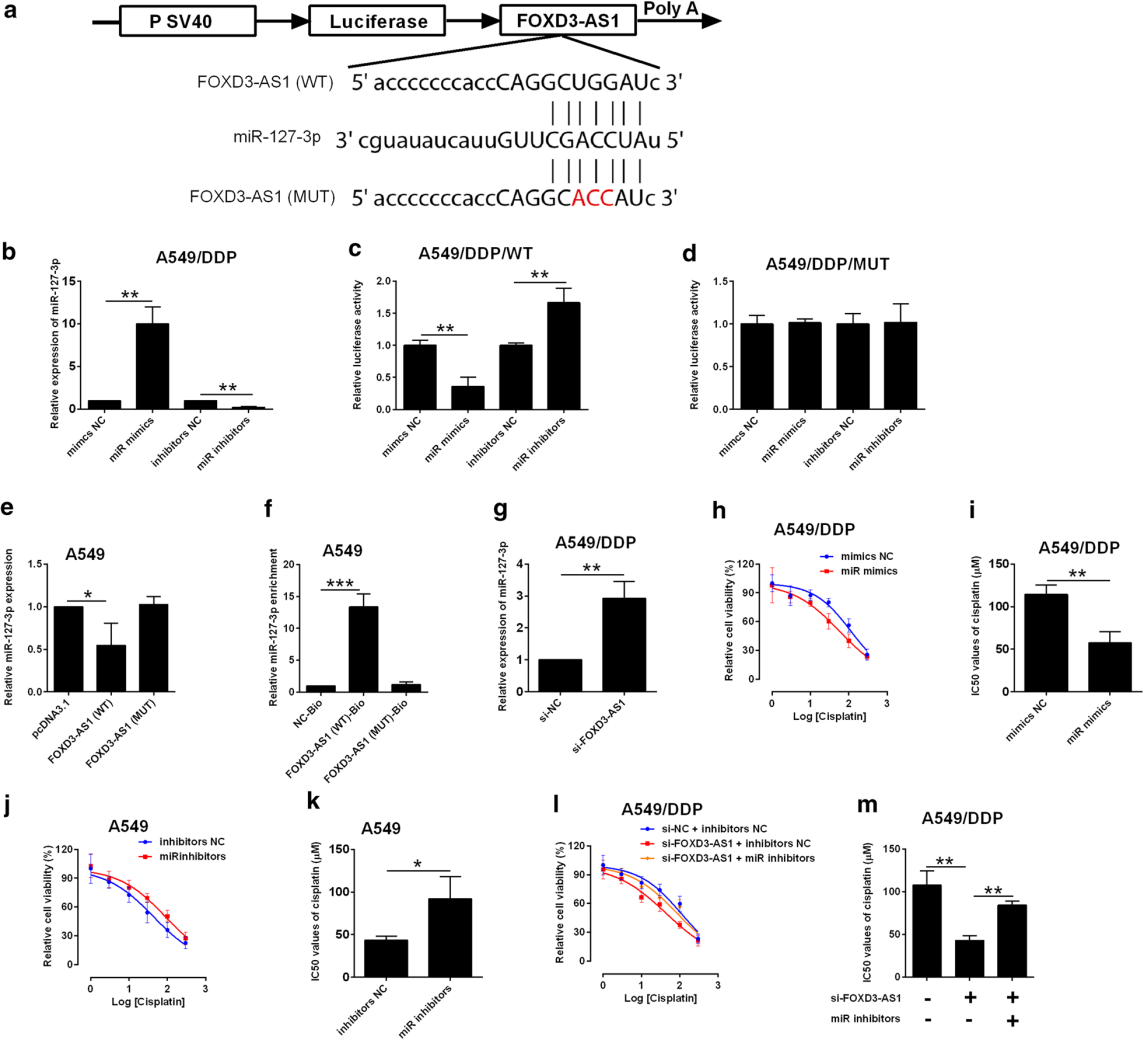
FOXD3-AS1 repressed miR-127-3p expression in NSCLC cells. **a** Predicted binding sites between FOXD3-AS1 fragments and miR-127-3p. WT = wild type; MUT = mutated. **b** qRT-PCR determination of miR-127-3p expression in A549/DDP cells with mimic NC, miR mimics, inhibitors NC or miR inhibitors transfection. **c**, **d** Dual-luciferase reporter assay analysis of relative luciferase activity of reporter vector containing wild type or mutated FOXD3-AS1 fragment in A549/DDP cells with mimic NC, miR mimics, inhibitors NC or miR inhibitors transfection. **e** qRT-PCR determination of miR-127-3p expression in A549 cells transfected with pcDNA3.1, pcDNA3.1-FOXD3-AS1 (WT) or pcDNA3.1-FOXD3-AS1 (MUT). **f** RNA pull-down assay determined the interaction between FOXD3-AS1 and miR-127-3p. **g** qRT-PCR determination of miR-127-3p expression in A549/DDP cells with si-NC or si-FOXD3-AS1 transfection. **h**, **i** CCK-8 assay determination IC50 values (cisplatin) in A549/DDP cells with mimics NC or miR mimics transfection. **j**, **k** CCK-8 assay determination of IC50 values (cisplatin) in A549 cells with inhibitors NC or miR inhibitors transfection. **l**, **m** CCK-8 assay determination of IC50 values (cisplatin) in A549/DDP cells after being transfected with different oligonucleotides. Mimics NC = negative control for miR-127-3p mimics; miR mimics = miR-127-3p mimics; inhibitors NC = negative control for miR-127-3p inhibitors); miR inhibitors = miR-127-3p inhibitors. N = 3 biological samples, and each sample was assayed in triplicates. Significant differences between groups were shown as *P < 0.05 and **P < 0.01

As of the ceRNA actions of lncRNAs, the miRNAs targeted by FOXD3-AS1 were extracted from the Starbase V3.0 datasets. MiR-127-3p was selected for further validation, as miR-127-3p was commonly predicted for its interaction with FOXD3-AS1 in several online algorithms. The luciferase activity was assessed in the luciferase reporter vectors containing FOXD3-AS1 fragments with miR-127-3p putative binding sites or its mutant fragment (Fig. 2a). MiR-127-3p overexpression (miR-mimics transfection) suppressed the relative luciferase activity of FOXD3-AS1 (WT); while miR-127-3p knockdown (miR-127-3p inhibitors transfection) had the opposite actions in A549/DDP cells (Fig. 2b, c). In contrast, changes in miR-127-3p expression was unable to influence luciferase activity of FOXD3-AS1 (MUT) (Fig. 2d). Overexpression of FOXD-AS1 (WT) suppressed miR-127-3p expression; whereas transfecting A549 cells with mutant FOXD3-AS1 vector had no effect on miR-127-3p expression (Fig. 2e). RNA pull-down assay further validated the interaction between FOXD3-AS1 and miR-127-3p (Fig. 2f). In a further confirmation, FOXD3-AS1 knockdown up-regulated miR-127-3p expression in A549/DDP cells (Fig. 2g). In the aspect of cisplatin-resistance, miR-127-3p overexpression sensitized the A549/DDP cells to cisplatin (Fig. 2h, i), while miR-127-3p knockdown promoted A549 cisplatin resistance (Fig. 2j–k). In a mechanistic aspect, the decreased cisplatin-resistance by FOXD3-AS1 knockdown was partially abolished by miR-127-3p inhibition in A549/DDP cells (Fig. 2l–m).

### MiR-127-3p repressed MDM2 expression in NSCLC cells

**Fig. 3:**
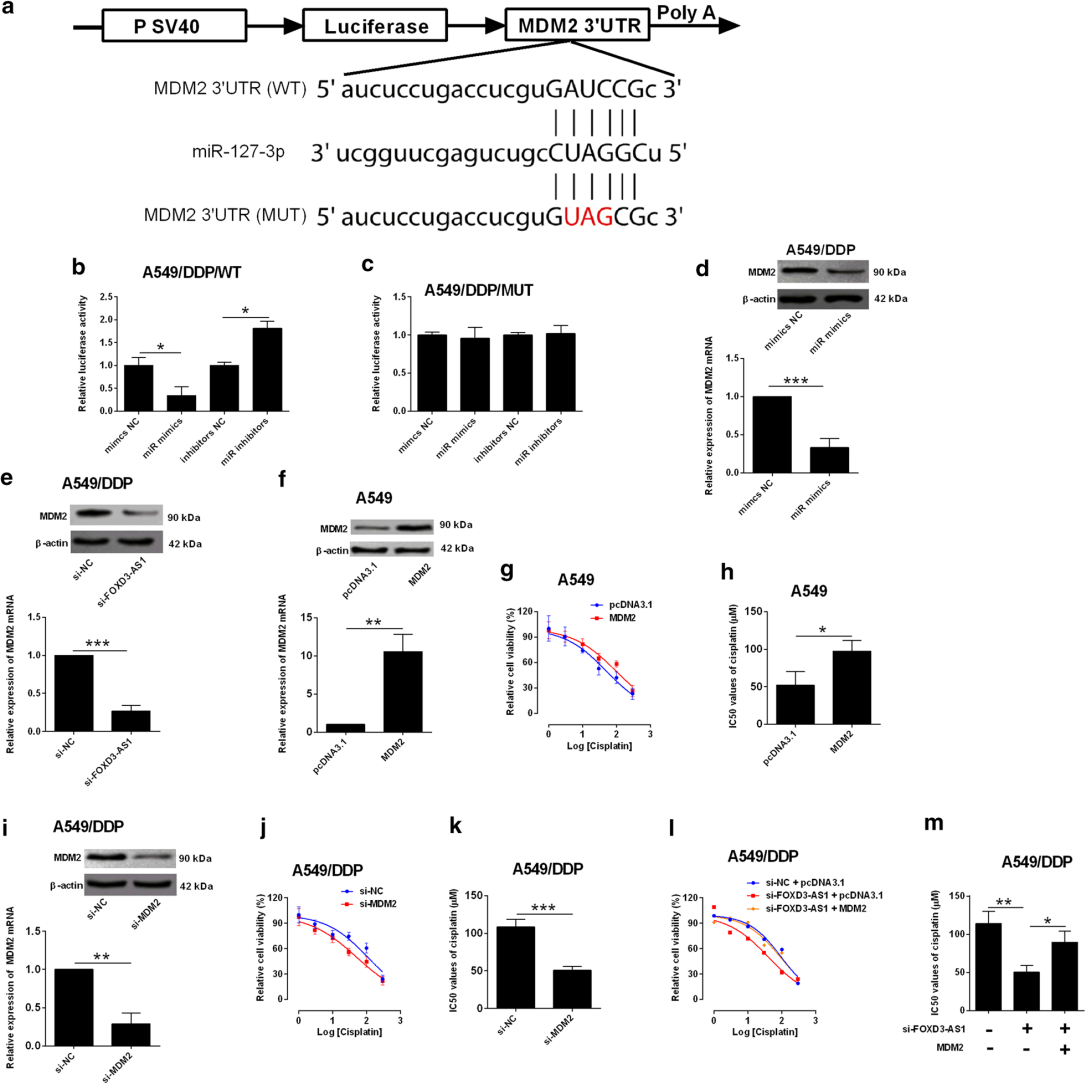
MiR-127-3p repressed MDM2 expression in NSCLC cells. **a** Predicted binding sites between miR-127-3p and MDM2 3′UTR. WT = wild type; MUT = mutated. **b**, **c** Dual-luciferase reporter assay analysis of relative luciferase activity of reporter vector containing wild type or mutated MDM2 3′UTR in A549/DDP cells with mimic NC, miR mimics, inhibitors NC or miR inhibitors transfection. **d** qRT-PCR and western blot analysis of MDM2 expression in A549/DDP cells with mimics NC or miR mimics transfection. **e** qRT-PCR and western blot analysis of MDM2 expression in A549/DDP cells with si-NC or si-FOXD3-AS1 expression. **f** qRT-PCR and western blot analysis of MDM2 expression in A549 cells with pcDNA3.1 or pcDNA3.1-MDM2 transfection. **g**, **h** CCK-8 assay analysis of IC50 values (cisplatin) in A549 cells with pcDNA3.1 or pcDNA3.1-MDM2 transfection. **i** qRT-PCR and western blot analysis of MDM2 expression in A549/DDP cells with si-NC or si-MDM2 transfection. **j**, **k** CCK-8 assay analysis of IC50 values (cisplatin) in A549/DDP cells with si-NC or si-MDM2 transfection. **l**, **m** CCK-8 assay analysis of IC50 values (cisplatin) in A549/DDP cells after being transfected with different oligonucleotides. Mimics NC = negative control for miR-127-3p mimics; miR mimics = miR-127-3p mimics; inhibitors NC = negative control for miR-127-3p inhibitors); miR inhibitors = miR-127-3p inhibitors. N = 3 biological samples, and each sample was assayed in triplicates. Significant differences between groups were shown as *P < 0.05, **P < 0.01 and ***P < 0.001

As miRNAs mediated the mRNA repression via targeting 3′UTR of targeting genes, predicted 3′UTRs with binding sites for miR-127-3p were analyzed with Starbase V3.0 database. MDM2 was selected from the predicted genes, as MDM2 was an important regulator for chemo-resistance. The luciferase activity was assessed in the luciferase reporter vectors containing MDM2 3′UTR fragments with miR-127-3p putative binding sites or its mutant fragment (Fig. 3a). MiR-127-3p overexpression (miR-mimics transfection) suppressed the relative luciferase activity of MDM2 3′UTR (WT); while miR-127-3p knockdown had the opposite actions in A549/DDP cells (Fig. 3b). In contrast, changes in miR-127-3p expression was unable to influence luciferase activity of MDM2 3′UTR (MUT) (Fig. 3c). In a further confirmation, miR-127-3p overexpression and FOXD3-AS1 knockdown both down-regulated MDM2 expression in A549/DDP cells (Fig. 3d, e). In the aspect of cisplatin resistance,MDM2 overexpression (Fig. 3f) promoted A549 chemo-resistance to cisplatin (Fig. 3g–h), while MDM2 knockdown (Fig. 3i) sensitized A549/DDP cells to cisplatin treatment (Fig. 3j–k). In a mechanistic aspect, the decreased chemo-resistance by FOXD3-AS1 knockdown was partially abolished by MDM2 overexpression in A549/DDP cells (Fig. 3l–m).

### FOXD3-AS1 regulated MPR1 expression via miR-127-3p/MDM2 axis

**Fig. 4:**
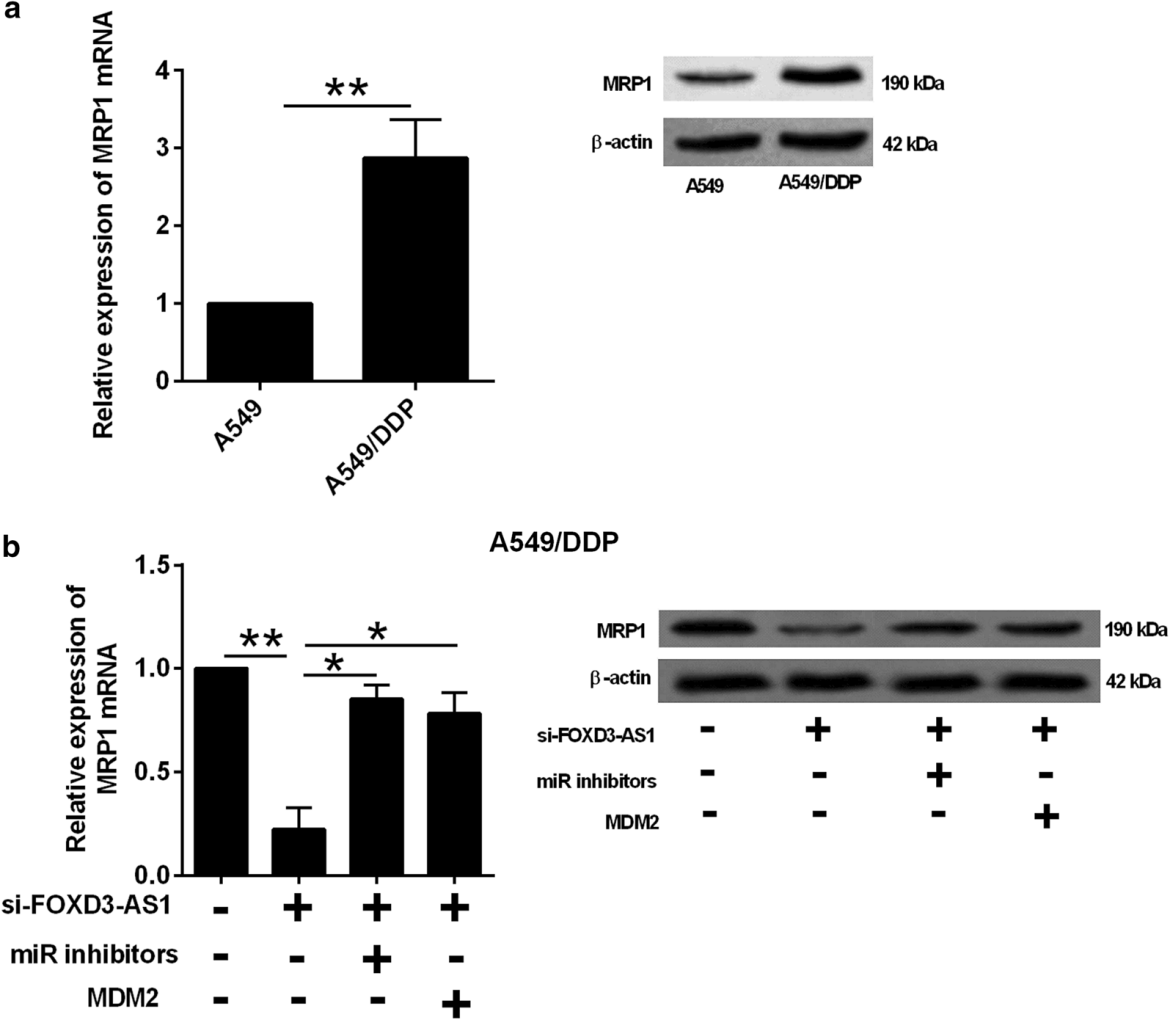
FOXD3-AS1 regulated MPR1 expression via miR-127-3p/MDM2 axis. **a** qRT-PCR and western blot analysis of MRP1 expression in A549 and A549/DDP cells. **b** qRT-PCR and western blot analysis of MRP1 expression in A549/DDP cells after being transfected with different oligonucleotides. miR inhibitors = miR-127-3p inhibitors. N = 3 biological samples and each sample was assayed in triplicates. Significant differences between groups were shown as *P < 0.05 and **P < 0.01

As MPR1 is a well-known gene that contributes to chemo-resistance, we further determined if this gene is involved in the regulatory network of FOXD3-AS1. The MRP1 expression was up-regulated in A549/DDP cells in comparison with its parental cell line (Fig. 4a). FOXD3-AS1 knockdown suppressed MPR1 expression in A549/DDP cells, which was partially counteracted by miR-127-3p knockdown or MDM2 overexpression (Fig. 4b).

### FOXD3-AS1 knockdown enhanced DDP sensitivity of A549/DDP cells in vivo

**Fig. 5:**
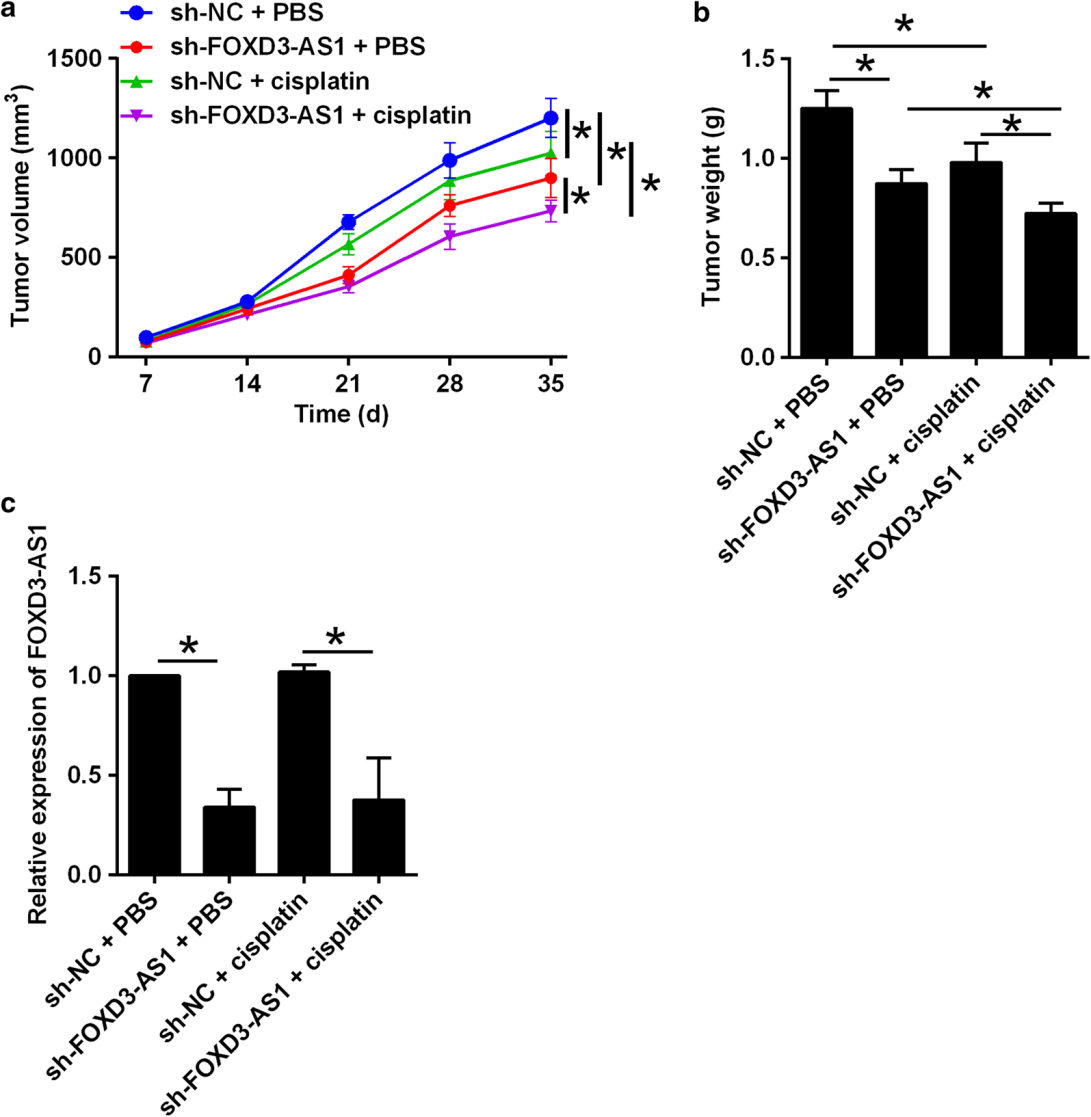
FOXD3-AS1 knockdown enhanced cisplatin sensitivity of A549/DDP cells in vivo. **a** Growth curve of xenograft tumors after different treatments. **b** Weights of dissected tumors in different groups. **c** qRT-PCR determination of FOXD3-AS1 expression in dissected tumor tissues in different groups. N = 6 biological samples, and each sample for FOXD3-AS1 expression was assayed in triplicates. Significant differences between groups were shown as *P < 0.05

As shown in Fig. 5a, FOXD3-AS1 knockdown suppressed the in vivo tumor growth of A549/DDP cells, and in the cisplatin-treated mice, FOXD3-AS1 knockdown caused a further suppression on the in vivo tumor growth of A549/DDP cells (Fig. 5a). The changes of tumor weight showed a consistent profile (Fig. 5b). The examination of tumor tissues by qRT-PCR showed that FOXD3-AS1 knockdown in A549/DDP cells down-regulated FOXD3-AS1 expression in the tumor tissues (Fig. 5c).

### Expressions of FOXD3-AS1, miR-127-3p and MDM2 in lung cancer tissues

**Fig. 6:**
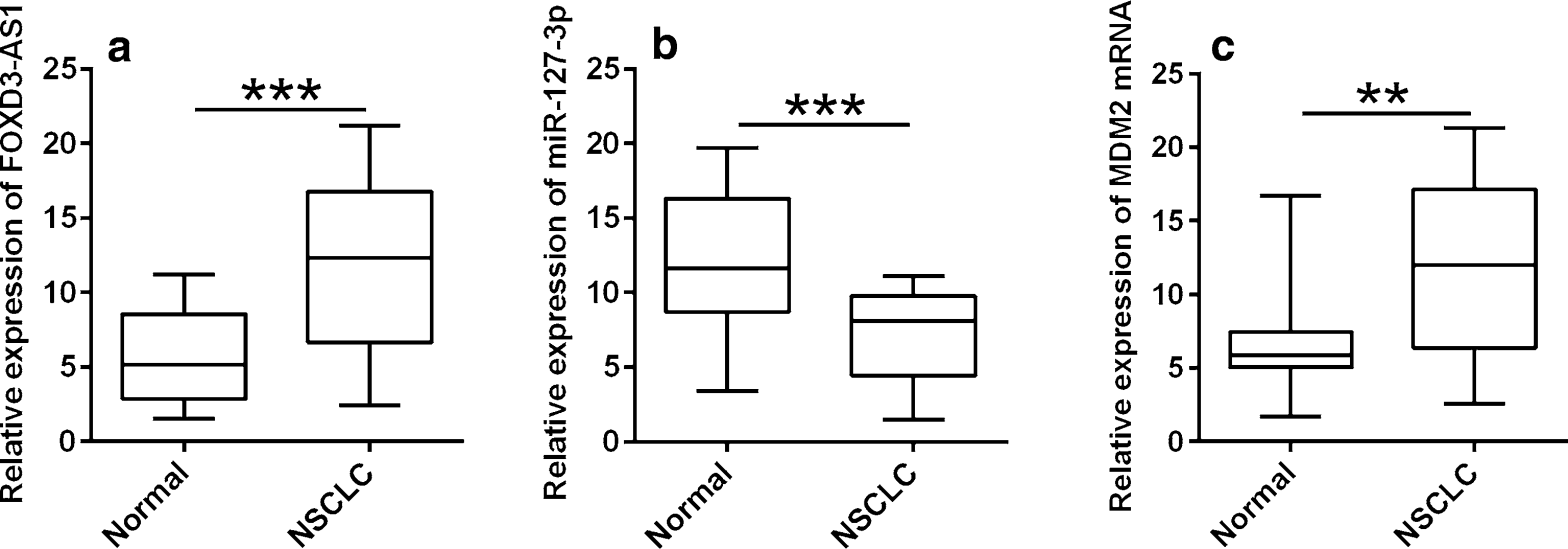
Expressions of FOXD3-AS1, miR-127-3p and MDM2 in lung cancer clinical tissues. qRT-PCR analysis of **a** FOXD3-AS1, **b** miR-127-3p and **c** MDM2 mRNA expressions in 40 lung cancer clinical tissues and adjacent normal lung tissues. N = 40 biological samples, and each sample was assayed in triplicates. Significant differences between groups were shown as **P < 0.01 and ***P < 0.001

The comparison for FOXD3-AS1, miR-127-3p and MDM2 expressions was performed in lung cancer tissues and normal adjacent lung tissues. As shown in Fig. 6, FOXD3-AS1 (Fig. 6a) and MDM2 mRNA (Fig. 6b) expression were up-regulated while miR-127-3p expression (Fig. 6c) was down-regulated in the lung cancer tissues in comparison with normal adjacent tissues.

## Discussion

The chemo-resistance is a great obstacle in the chemotherapy treatment for NSCLC [23, 24]. Hence, development of novel targets to potentiate chemo-sensitivity is essential to improve the clinical outcomes of NSCLC patients. Recently, existing evidence implied that lncRNAs play vital roles in NSCLC progression and chemo-resistance. In the present study, FOXD3-AS1 was up-regulated in NSCLC cells with cisplatin-resistance i.e. A549/DDP and H1299/DDP cells in comparison with the parental NSCLC cells. Moreover, lung cancer clinical tissues also displayed higher FOXD3-AS1 expression than adjacent normal lung tissues. This study first investigated the enhanced effects of FOXD3-AS1 on cisplatin resistance in NSCLC and verified the interaction between FOXD3-AS1 and miR-127-3p in NSCLC cells.

As far as we know, the role of FOXD3-AS1 in malignant tumor progression has been deciphered in several types of cancers. FOXD3-AS1 knockdown impaired glioma cell proliferation and metastasis, suggesting the oncogenic role of FOXD3-AS1 in glioma progression [12]. FOXD3-AS1 was found to be up-regulated in breast malignant tumor and was correlated with advanced clinical stages, and silence of FOXD3-AS1 significantly attenuated the breast cancer cell proliferation and metastasis [13]. FOXD3-AS1 was abundantly expressed in both colorectal cancer (CRC) tissues and cells, FOXD3-AS1 overexpression was associated with worse clinical outcomes of CRC patients. In addition, silence of FOXD3-AS1 was effective in suppressing CRC cell progression [15]. In this study, we first showed that FOXD3-AS1 was up-regulated in A549/DDP and H1229/DDP cells compared to their corresponding parental NSCLC cells. Gain-of-function assays showed that FOXD3-AS1 increased the IC50 values of cisplatin in A549 and H1299 cells; while FOXD3-AS1 knockdown decreased IC50 values of cisplatin in A549/DDP and H1299/DPP cells, suggesting that FOXD3-AS1 exerts promoting effects on cisplatin resistance in NSCLC cells.

One of the main functions of lncRNA is known to act as a ceRNA to influence microRNAs (miRNAs) expression. Using Starbase V3.0 tool, miR-127-3p was predicted to target the MDM2 3′UTR. Luciferase reporter assay confirmed the interaction between FOXD3-AS1 and miR-127-3p. So far, the role of miR-127-3p has been uncovered in several types of malignant tumors. MiR-127-3p was down-regulated in osteosarcoma tissues and cell lines and acted as a tumor-suppressor in osteosarcoma [20, 25]. The tumor suppressive effects of miR-127-3p was also uncovered in ovarian cancer [19]. Down-regulation of miR-127-3p was identified in oral squamous cell carcinoma, and miR-127-3p suppressed this malignant tumor progression [26]. Our results revealed that miR-127-3p overexpression sensitized A549/DDP cells to cisplatin treatment; while miR-127-3p inhibition promoted chemo-resistance in A549 cells. Moreover, the suppressive effects of FOXD3-AS1 on chemo-resistance were abolished by miR-127-3p inhibition in A549/DDP cells. These results may suggest that FOXD3-AS1 regulates chemo-sensitivity of NSCLC cells via sponging miR-127-3p.

Further investigation into the downstream signaling of miR-127-3p revealed that MDM2 was repressed by miR-127-3p in NSCLC cells. In addition, MDM2 was down-regulated in A549/DDP cells by FOXD3-AS1 siRNA transfection. MDM2 is part of a negative feedback loop in which p53 acts as a transcription factor for MDM2. MDM2 overexpression could lead to malignant transformation of the cell [27–29]. Inhibition of MDM2 was found to sensitize the NSCLC cells to cisplatin via promoting apoptosis [27]. In addition, inhibition of MDM2 was found to sensitize NSCLL cells to radiotherapy via promoting premature senescence induced by radiation [30]. In the present study, we revealed that MDM2 overexpression promoted chemo-resistance of A549 cells and counteracted the suppressive effects of FOXD3-AS1 knockdown on chemo-resistance; while MDM2 knockdown sensitized A549/DDP to cisplatin. Moreover, FOXD-AS1 knockdown suppressed MRP1 expression which was attenuated by miR-127-3p knockdown and MDM2 overexpression. In in vivo studies further confirmed that FOXD3-AS1 suppressed in vivo tumor growth of A549/DDP cells. Clinical investigations showed the up-regulation of FOXD3-AS1 and MDM2; while down-regulation of miR-127-3p in treatment-sensitive lung cancer tissues. All in all, these results implied that FOXD3-AS1 regulated cisplatin resistance of NSCLC cells via modulating miR-127-3p/MDM2 axis.

MRP1 belongs to the ATP-binding cassette transporter superfamily and can be divided into 7 distinct subfamilies; and various studies have shown that MPR1 involved in cisplatin-induced multidrug resistance [31, 32]. Our studies indicated that FOXD3-AS1-mediated cisplatin resistance may be associated with MRP1. In fact, MRP1 can be regulated various several lncRNAs such as HOX antisense intergenic RNA, metastasis associated lung adenocarcinoma transcript 1, plasmacytoma variant translocation 1 and differentiation antagonizing non-protein coding RNA across different types of cancers [33]. However, the current study lacks the information linking MDM2 and MRP1, which should be considered in the future directions. FOXD3 has been found to act as a tumor suppressor in lung cancer [34], while the interaction between FOXD3 and FOXD3-AS1 has not been elucidated in this study, which may require further studies. Another limitation of our study is that the mechanistic investigation is limited to A549 cells, and further studies may be needed to perform in H1299 cells to further confirm our findings.

## Conclusion

**Fig. 7:**
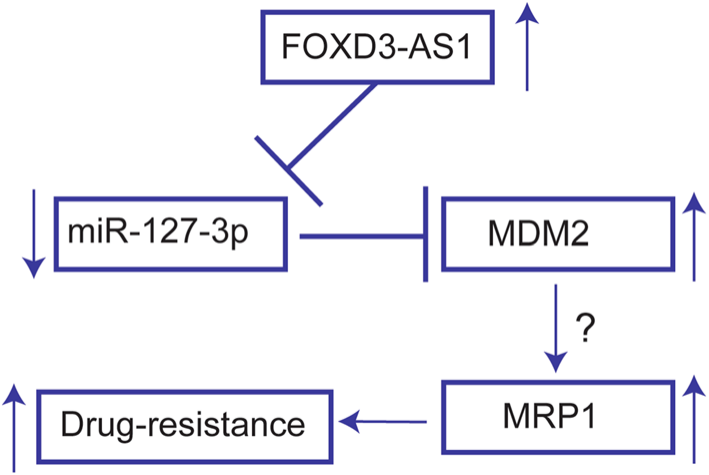
Proposed mechanistic actions of FOXD3-AS1 in regulating drug resistance in lung cancer cells

In conclusion, our study demonstrated the pivotal roles of FOXD3-AS1/miR-127-3p/MDM2 regulatory signaling pathways in cisplatin resistance of NSCLC cells (Fig. 7). The present study may provide new insights into overcoming cisplatin resistance in NSCLC treatment.

## Supplementary information

**Additional file 1: Table S1.** Sequences for oligonucleotides.

## Data Availability

All the datasets in the manuscript are available.

## Abbreviations

3′UTR: 3′ Untranslated region
BLCAT1: Bladder cancer associated transcript 1
CCAT1: Colon cancer associated transcript 1
CCK-8: Cell counting kit-8
ceRNA: Competing endogenous RNA
CRC: Colorectal cancer
EGFR-AS1: EGFR antisense RNA 1
FBS: Fetal bovine serum
FOXD3-AS1: FOXD3 antisense RNA1
lncRNAs: Long non-coding RNAs
miRNA: MicroRNA
MRP1: Multidrug resistance protein 1
MUT: Mutant
MDM2: Murine double minute 2
NHBE: Normal human lung bronchial epithelial cell
NSCLC: Non-small cell lung cancer
siRNA: Small interfering RNA
