# Supplementary material for: LncRNA FOXD3-AS1 promoted chemo-resistance of NSCLC cells via directly acting on miR-127-3p/MDM2 axis
Source: Cancer Cell Int. 2020 Jul 29;20:350. doi: 10.1186/s12935-020-01402-9 (PMC7388492; doi:10.1186/s12935-020-01402-9)
Supplement: Supplementary file 1 — Additional file 1: Table S1. Sequences for oligonucleotides. [file 12935_2020_1402_MOESM1_ESM.docx]

**Table S1. Sequences for oligonucleotides**

| Oligo set | Sequences |
| --- | --- |
| si-NC for FOXD3-AS1 | 5’-CCTCCAGTGACCGCCTAAG-3’ |
| si-FOXD3-AS1 | 5’-GCTACTTGGAGTTGTTAAA-3’ |
| si-NC for MDM2 | 5’-GGGCCAGGCTGCTAACGCACATGTCATA-3’ |
| si-MDM2 | 5’-CGTCGCGAGGGCTATGAACTAATGACCC-3’ |
| sh-FOXD3-AS1 | 5’AGGGATACAAGCATATACCACTCGAGTGGTATATGCTTGTATCCCTC-3' |
| sh-FOXD3-AS1 | 5'GATCCCGTGTGGACAAATCCTCCAAGACTCGAGTCTTGGAGGATTTGTCCACACTTTTTGGAT-3' |
| Mimics NC | 5’- GUCCUCGGUGUGACCUGCUAUG-3’ |
| miR-127-3 mimics | 5’-UCGGAUCCGUCUGAGCUUGGCU-3’ |
| Inhibitors NC | 5’- GCCAAUCGCUGCCAAGAUUCCGACGCCUA-3’ |
| miR-127-3p inhibitors | 5’-AGCCAAGCUCCUAGGAUCCGACUUCAGCC-3’ |
